# Occurrence of the specific long spike burst pattern in the ovine proximal gallbladder as an indication of myoelectric regional variability

**DOI:** 10.4102/ojvr.v85i1.1455

**Published:** 2018-06-11

**Authors:** Krzysztof W. Romański, Józef Nicpoń

**Affiliations:** 1 Centre for Experimental Diagnostics and Biomedical Innovations, Wrocław University of Environmental and Life Sciences, Poland

## Abstract

The myoelectrical activity of the ovine gallbladder has not been fully recognised. Five rams were fitted with six small intestinal and three gallbladder electrodes and a strain gauge force transducer was mounted near the gallbladder fundic electrode. In two series of successive experiments, the electromyographical and mechanical recordings were recorded over a period of 5–7 hours. The occurrence of the slow waves in the small bowel was regular, unlike those in the gallbladder. In the gallbladder infundibulum, the specific pattern, called the long spike burst pattern (LSBP), was observed. It comprised usually one or two parts of prolonged duration. The first part resembled the classical (short lasting) spike burst in the small bowel, and its amplitude was lower than that of the second part. The spike burst frequency of the second part was 2–3 times lower than that of the first part. During phase 1-like and phase 2a-like activities, the intensity of the gallbladder LSBP was reduced while enhanced after feeding. In fasted rams, the duration of a specific pattern, observed in the gallbladder infundibulum, was longer than in non-fasted animals and its amplitude was low. Similar events were recorded in the gallbladder corpus, but the specific pattern was shorter and irregular. In the gallbladder fundus, mostly irregular short spike bursts were recorded. It is concluded that in sheep, specific types of the long-lasting groups of spikes occur in the upper gallbladder areas exhibiting myoelectrical regional variability. The character of an LSBP depends on feeding conditions.

## Introduction

Sheep belong to the animal species in which the biliary tract contains well-developed gallbladders. Its size is similar to that in man and dog; thus, it may store a substantial portion of the bile, and inflow and outflow of bile is almost continuous (Aziz & Khatra 1985). The gallbladder, similar to the small bowel, exhibits intense motor functions representing both myoelectrical and mechanical activities. The slow and rapid action potentials as the periodic deflections of the resting membrane potential can be distinguished (Szurszewski 1987). Slow waves are generated by at least some types of the interstitial cells of Cajal (ICC) (Thuneberg 1999), the gastrointestinal pacemaker cells, because of slow transmembrane ion movements. Interstitial cells of Cajal along with the smooth muscle cells (SMC) and with the cells expressing the platelet-derived growth factor receptors *α*^+^ (PDGFR*α*^+^ cells) form SMC/ICC/PDGFα^+^ syncytium (SIP syncytium) responsible for pacemaker activity and provide propagation pathways for the slow waves (Sanders, Ward & Koh 2014). Usually, the slow waves (even at the peak of the upstroke phase) cannot reach the critical voltage threshold to evoke the rapid action potential (spike) responsible for mechanical activity. The spikes are usually organised in the small groups, namely the spike bursts, and they are restricted, in most cases, to the plateau phase of the slow waves. The spike bursts are omnipresent in the stomach and small bowel, and a similar situation exists in the gallbladder (Buéno & Praddaude 1979; Szurszewski 1987). The spike bursts of longer durations, that is, which last longer than the plateau phase of the slow wave (over 3 sec in the stomach), can occur independently of the slow waves and induce longer contractions (Sarna 2002). Slow waves arrive in the stomach and small bowel regularly, also in sheep (Roman´ski 2002a). They are also present in the ovine gallbladder, although not always detected there (Roman´ski 2004a). Thus, the ovine gallbladder exhibits intense motor activity and plays an important role in bile circulation (Pass & Heath 1977). The principal physiological roles of the gallbladder are to concentrate and excrete bile (Lee & Kuwer 2006; Shaffer 2000). Gallbladder emptying is more efficient after a meal, but during the fasting state periodic gallbladder contractions also evacuate bile into the duodenum (Dodds, Hogan & Geenen 1989; Ryan 1981; Shaffer 2000). Therefore, its motor function must be precise enough to ensure filling and emptying through the common bile duct as well as bile storage and mixing to prevent sedimentation of bile constituents. Gallbladder motility also facilitates bile concentration. Furthermore, the incessant but fluctuating motor activity of the gallbladder must be coordinated with that of the small bowel. In ruminants, there are no clear interdigestive periods, and Ruckebusch (1989) suggested that periodic gallbladder contractions are also present, at least in sheep. Therefore, the presence of specific motility patterns in the gallbladder can be expected, also in sheep (Kaji, Takamatsu & Kojiya 2002). It was already reported that in sheep the migrating myoelectric complex (MMC)-like activity may occur in the gallbladder, as it was observed in the small bowel, and the ‘minute rhythm’ (MR) regularly arrived both in the small bowel and the gallbladder during its phase 2 activity (Grivel & Ruckebusch 1972; Roman´ski 1996, 2002b, 2017; Ruckebusch 1989). Thus, the ovine gallbladder motility exhibits some similarities to the small intestinal motility. It seems likely that the regional differences in ovine gallbladder myoelectrical activity can also occur (Buéno & Praddaude 1979; Roman´ski 2004a). Therefore, the aim of this study was to characterise further the gallbladder motility in sheep under various feeding conditions with special emphasis directed towards the motility patterns and regional differences.

## Materials and methods

Five healthy rams of Polish Merino breed, weighing between 38 kg and 43 kg, were used. Before surgery, the animals were kept in large cages under normal day–night lighting conditions and fed good quality hay together with grain mixture. Drinking water was not limited.

### Animal preparation

In the 24 h fasted rams, the general and local anaesthesia was applied just prior to surgery. A right-sided lateral laparotomy was performed. Each ram was fitted with nine bipolar platinum wire electrodes for electromyographical recordings, and one strain gauge force transducer was attached for mechanical recordings. The teflon-covered electrodes (made by us) and the strain gauge force transducers embedded in rubber-teflon (Silastic-type material) coat (RB Products, Madison, WI) were sutured to the serosal side of the gastrointestinal and gallbladder walls. Electrode localisation: (1) the duodenal bulb, 6 cm distal to the middle of the pyloric ring; (2) the duodenum, 56 cm distal to the middle of the pyloric ring; (3) proximal jejunum, 200 cm distal to the duodenal electrode; (4) more distal jejunum, 100 cm distal to the first jejunal electrode; (5) subterminal ileum, 110 cm proximal to the middle of the ileocecal junction; (6) terminal ileum, 10 cm proximal to the middle of the ileocecal junction; (7) gallbladder infundibulum, about 1 cm distal to the cystic duct; (8) gallbladder corpus, 4 cm distal to the proximal gallbladder electrode; and (9) gallbladder fundus, 4 cm distal to the mid gallbladder electrode. The small strain gauge force transducer was attached next to the distal gallbladder electrode. It was used for additional confirmation of the correctness of myoelectrical recordings and for the assessment of types of gallbladder fundic contractions. The marked electrode and transducer wires were exteriorised over the skin, soldered to the plugs and fixed 2 cm from the skin incision. They were connected with the recording apparatus each time during the experimental periods and disconnected afterwards. After the surgery, rams were allowed at least two weeks to recover.

### Experiments

A total of 30 timed experiments, each lasting for 5–7 h, were conducted. Four different conditions were considered. The first two were the experiments performed on 48 h fasted animals, with or without feeding during the experiment. Fed animals received 250 g of a grain mixture which they consumed over 3–4 min. Food was given during the late phase 2 of the duodenal MMC (and possibly the gallbladder), at about 70% of the MMC cycle. During further experiments performed in fasted and non-fasted animals, food was offered during the early phase 2 of the duodenal MMC (gallbladder MMC-like activity occurred concurrently with that in the duodenum), at about 30% of the MMC cycle. Each experiment was conducted on each ram. The 10-channel electroencephalograph (Reega Duplex TR XVI), adapted for mechanical recordings, was used in the study. The myoelectrical and motor activities were recorded from nine electrodes and from the strain gauge force transducer. The small intestinal myoelectrical recordings serving for MMC and MR recognition in the small bowel were also helpful for interpretation of the gallbladder motility recordings. At least two full MMC cycles were recorded during each experiment. Other details of the methodology are described elsewhere (Roman´ski 2004a, 2016).

### Data elaboration

In the small-intestinal recording sites, the regular occurrence of the MMC cycles was observed. In the gallbladder, the presence of MMC-like activity was also detected, as suggested previously (Roman´ski 1996). Particular attention was given to phase 2 of the gallbladder MMC-like pattern. To characterise it, at least in part, several parameters were calculated and presented mostly in tables. They contained the spike bursts of different duration, especially the short spike bursts (classical, i.e., the myoelectrical correlates of shorter phasic contractions) and the long-lasting spike bursts. The classical spike burst frequency, amplitude, duration, number of spikes in one burst and number of spikes per second were calculated for the spike bursts noticed in all the gallbladder regions examined. Similar parameters were calculated for the longer myoelectrical events. These episodes were observed in the upper gallbladder regions, namely in the gallbladder infundibulum and gallbladder corpus. They were called ‘the long spike burst patterns’ (LSBPs), because the individual spikes were less frequent than those in the short spike bursts. The frequency, amplitude and duration of fundic longer and shorter contractions were calculated as well.

### Statistics

The mean values and standard deviations were calculated from the raw data. Finally, the Student’s *t*-test for paired values, preceded by the analysis of variance, was applied (Snedecor & Cochran 1971). The statistical significance was labelled when *p* < 0.05.

### Ethical consideration

The experimental model used in this article was approved by the II Local Ethical Committee for Experimental Animals in Wrocław, Poland. Therefore, all the experiments were performed officially, according to Polish and European laws.

## Results

In the small bowel, typical MMC cycles and MR episodes (both are not shown) were identified in the recordings. In the gallbladder, the pattern resembling the MMC in the small bowel occurred in a cyclical fashion. In both upper gallbladder regions, phase 1 of the MMC-like activity was less clear than that in the gallbladder fundus because it was interrupted by regularly arriving LSBPs. Phase 2 of the MMC arrived in the duodenum and the phase 2-like activity was seen in the gallbladder similar to that in the duodenum. The short spike bursts (the myoelectrical correlates of typical phasic contractions) were observed throughout this phase both in the small bowel and the gallbladder. The short spontaneous spike bursts arriving in the course of phase 2 of the gallbladder MMC-like activity (except the spike bursts forming the gallbladder MR, as seen in Figure 2) are shown in Table 1. The duration of the spike burst and the number of spikes in one spike burst were slightly greater in fed rams than in non-fed rams. The spike burst amplitude was significantly lower in fed animals than in non-fed animals and was slightly lower in the gallbladder fundus as compared with that in the gallbladder infundibulum. The frequency of the spike bursts was greater in fed rams than in fasted rams and especially higher in the gallbladder fundus and lowest in gallbladder infundibulum. The spike frequency measured within the spike burst was higher in fed rams than in non-fed rams, particularly in the experiments performed in non-fasted animals. In the gallbladder fundus, these values were slightly greater compared to other gallbladder regions examined (Table 1).

**TABLE 1:** Characteristics of the short spike bursts recorded during phase 2-like activity of the gallbladder migrating myoelectric complex as observed in the gallbladder infundibulum, corpus and fundus in various feeding conditions.

Fasting	Feeding	Mean ± S.D.	Duration(s)	Amplitude (*μ*V)	Spike burst frequency (cpm)	Number of single spikes	Spike frequency (No/s)
Fasted	Non-fed	Mean I	1.1	40.4	1.4	6.3	5.1
		± SD	0.3	8.9	0.5	0.4	1.2
		Mean C	0.8	35.9	3.8	4.9	5.0
		± SD	0.5	11.3	0.7	0.3	0.9
		Mean F	0.9	29.4	7.2	5.8	5.3
		± SD	0.3	8.6	0.6	0.5	1.0
	Fed	Mean I	1.4	25.3*	2.5*	7.7**	7.9*
		± SD	0.3	7.2	0.7	0.5	2.0
		Mean C	1.3*	20.7	9.1***	7.2**	7.3
		± SD	0.2	8.4	2.9	0.7	2.2
		Mean F	1.3	22.3	10.4*	6.8*	8.6*
		± SD	0.3	6.6	1.9	0.5	2.3
Non-fasted	Non-fed	Mean I	1.0	34.7	1.8†	5.4†	6.6
		± SD	0.4	6.4	0.4	0.4	2.7
		Mean C	1.1	32.5	4.1	5.8†	6.2
		± SD	0.3	7.3	2.3	0.4	3.1
		Mean F	0.9	31.2	11.7†	5.1	6.8
	Fed	± SD	0.3	8.2	2.8	0.6	2.8
		Mean I	1.2	21.6*	2.6	7.7***	9.4
		± SD	0.3	7.4	1.3	0.6	3.3
		Mean C	1.2	21.3*	7.0*	7.9***	10.5
		± SD	0.2	5.8	0.6	0.5	3.2
		Mean F	1.1	18.7*	14.5	8.1***†	11.1*
		± SD	0.3	6.4	3.9	0.7	1.7

Note: Number of single spikes – number of single spikes present in one spike burst. Other explanations are presented in the ‘Materials and methods’ section.

*n* = 5.

*μ*V, calibration; cpm, cycles per minute; I, gallbladder infundibulum; C, gallbladder corpus; F, gallbladder fundus.

* , *p* < 0.05;

** , *p* < 0.01;

*** , *p* < 0.001 (Statistical significances versus relevant ‘non-fed’ value.).

† , *p* < 0.01 (Statistical significances versus relevant ‘fast’ value).

**FIGURE 1:**
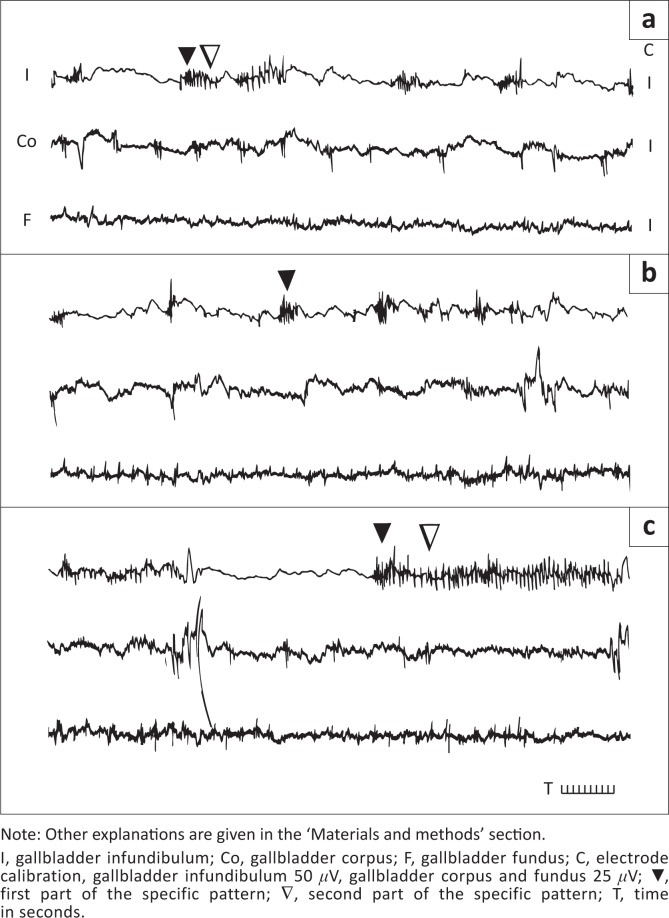
Three fragments of the gallbladder myoelectrical recording in fasted rams. (a) Recording during phase 1 of the migrating motility complex observed both in the duodenum (3 min after the onset of duodenal phase 1) and the gallbladder; (b) recording during phase 2a of the migrating motility complex observed both in the duodenum (14 min after the onset of duodenal phase 1) and the gallbladder; and (c) recording during phase 2b of the migrating motility complex observed both in the duodenum (25 after the onset of duodenal phase 1) and the gallbladder.

**FIGURE 2:**
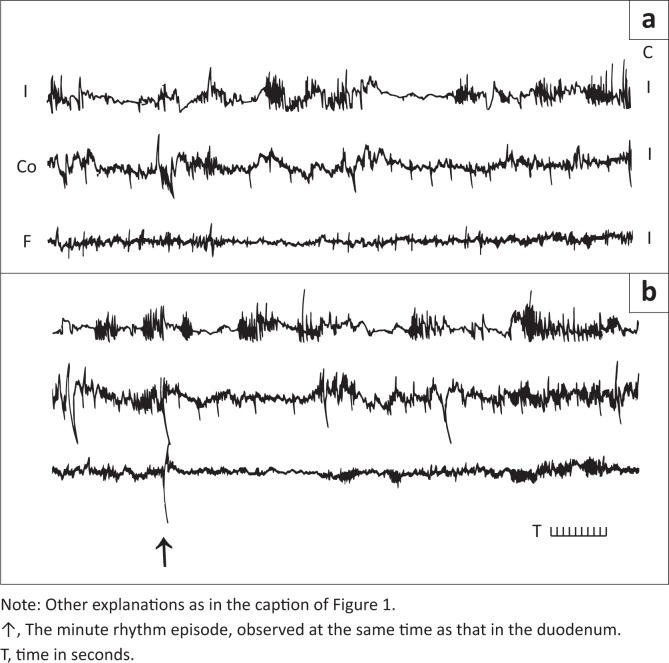
Two fragments of gallbladder myoelectrical recordings in fasted rams after feeding. (a) The recording started 9 min after termination of feeding and (b) continued recording after feeding.

The slow waves were regularly observed in the small bowel. They were observed occasionally in the gallbladder. Both amplitude and frequency of the slow waves were lower than the spikes of the second part of the LSBP (Figure 2). They can be seen in the gallbladder fundus (Figure 1) and in the gallbladder corpus and fundus (Figure 2a).

In the gallbladder infundibulum of fasted and non-fasted rams, the short spike bursts occurred irregularly along with the LSBP pattern (Table 2, Figure 1). They appeared regularly, regardless of the MMC phase. Long spike burst patterns arriving in the course of phase 2 of the gallbladder MMC-like activity usually contained two parts. The initial (shorter) part of higher spike frequency (Table 2, Figure 1) was observed in most cases at the beginning of LSBP. This part closely resembled typical prolonged (i.e. lasting longer than 2 sec) spike bursts. Occasionally, the first part of the LSBP occurred without the second part. This part of the pattern (longer duration and usually higher amplitude) contained less frequent spikes resembling rather the long spike bursts described in the ovine colon (Fioramonti & Hubert 1980). The occurrence of the LSBP in the gallbladder infundibulum was regular and its absence in this region was very exceptional. In the gallbladder corpus, LSBPs were shorter and absent more frequently than those in the gallbladder infundibulum (Table 2). The LSBP present in the gallbladder corpus contained two parts in about 85% of episodes. In 10% of episodes, at the end of each pattern, more frequent spike burst again arrived. This was occasionally observed in the gallbladder infundibulum. The first part of the LSBP evolved into the second one abruptly or gradually, directly or through the transient short and irregular fragment distinct from both parts of the pattern. In about 5% of LSBPs, their first part arrived again in the middle of the second part of the pattern. No LSBP was detected in the gallbladder fundus and in the small bowel.

**TABLE 2:** Characteristics of the long spike burst pattern recorded in the gallbladder infundibulum and corpus in various feeding conditions.

Fasting	Feeding	Mean ± S.D.	Duration (s)		Amplitude (*μ*V)		Frequency (cpm)		Spikes (N)	
			Infund.	Corp.	Infund.	Corp.	Infund.	Corp.	Infund.	Corp.
Fasted	Not fed	Mean	61.4	19.6	25.4	18.2	1.0	1.1	24.3	18.2
		± SD	14.1	8.3	6.5	7.4	0.4	0.4	5.9	3.4
	Fed	Mean	33.2**	13.6	57.3**	31.4*	2.8***	2.3**	35.8*	25.4
		± SD	9.4	4.6	18.4	9.2	0.4	0.5	6.4	4.2
Not fasted	Not fed	Mean	36.1‡	17.4	32.6	22.5	3.1§	2.6§	32.6	27.3
		± SD	7.3	7.0	7.1	9.7	0.8	0.7	5.3	3.9
	Fed	Mean	24.8*	12.7	47.3	33.0	4.6*§	3.4†	31.2	25.9
		± SD	4.2	7.5	11.3	12.2	0.9	0.8	9.8	4.4

Note: Other explanations are the same as in Table 1 and are also presented in the section ‘Materials and methods’.

*μ*V, calibration; cpm, cycles per minute; LSBP, long spike burst pattern; Spikes (N), number of spikes in the whole LSBP; infund., gallbladder infundibulum; corp., gallbladder corpus.

* , *p* < 0.05;

** , *p* < 0.01;

*** , *p* < 0.001 (Statistical significances versus relevant ‘non-fed’ value).

† , *p* < 0.05;

‡ , *p* < 0.01. (Statistical significances versus relevant ‘fast’ value).

§ , *p* < 0.001 (Statistical significances versus relevant ‘fast’ value).

In the gallbladder infundibulum during phase 1 of MMC-like activity observed in fasted rams, the LSBP duration was 13 ± 6 sec with amplitude of 64 *μ*V ± 18 *µ*V in the gallbladder infundibulum. During phase 1 in non-fasted rams, the respective values were 9 ± 4 sec and 67 *μ*V ± 20 *μ*V. In the gallbladder corpus, the LSBPs were less frequent and reduced in length.

The LSBP occurred in both feeding regimes designed in this study. The LSBPs observed during phase 2 of the gallbladder MMC-like activity were longer and developed further in the gallbladder infundibulum than in the gallbladder corpus regardless of feeding conditions (Table 2). The duration of the whole pattern was the longest during the fasting period. As the amplitude was measured in the middle of the pattern, these values characterised exclusively its longer (second) part. Both the amplitude and frequency of LSBP were the greatest in the experiments with feeding. In the gallbladder infundibulum and corpus, the number of spikes in the whole pattern was lower in the experiments performed on fasted rams than in the remaining experimental groups (Table 2).

In the gallbladder corpus, the duration of the first fragment of the LSBP was shorter than that in the gallbladder infundibulum (Table 3). In both these gallbladder regions, the values were greater in fasted animals after feeding compared to non-fed animals. The amplitude of these spike bursts was lower in the gallbladder corpus than in gallbladder infundibulum and was the lowest after feeding. The frequency of spikes forming the first part of the LSBP was the lowest in fasted animals and feeding increased this value. The number of spikes in the shorter fragment of LSBP was not significantly smaller in the gallbladder corpus than in gallbladder infundibulum. Similar differences of these values were observed between fed and non-fed animals (Table 3, also see Figure 2).

**TABLE 3:** Characteristics of the shorter fragment of long spike burst pattern recorded in the gallbladder infundibulum and corpus in various feeding conditions.

Fasting	Feeding	Mean ± S.D.	Duration (s)		Amplitude (*μ*V)		Frequency (cpm)		Spike (N)	
			Infund.	Corp.	Infund.	Corp.	Infund.	Corp.	Infund.	Corp.
Fasted	Non- fed	Mean	2.7	1.8	18.3	10.4	2.4	3.1	12.4	7.9
		± SD	0.4	0.7	2.2	2.0	0.3	0.4	4.6	3.3
	Fed	Mean	6.9***	2.7	12.6**	6.4**	3.0*	2.9	8.3	5.5
		± SD	1.1	1.2	1.8	0.8	0.4	0.6	2.7	2.0
Non-fasted	Non-fed	Mean	5.3§	3.4†	14.5	8.1	3.5†	4.8†	9.9	7.1
		± SD	0.8	0.9	2.2	1.2	0.6	1.5	3.4	2.6
	Fed	Mean	6.4	3.9	11.7	5.4	4.1†	5.9†	6.4	3.8
		± SD	1.0	0.6	1.8	0.6	0.5	2.3	1.1	2.2

Note: Other explanations are as in Tables 1 and 2, and also presented in the ‘Materials and methods’ section.

*μ*V, calibration; cpm, cycles per minute; LSBP, long spike burst pattern; Spikes (N), number of spikes in the whole LSBP; infund., gallbladder infundibulum; corp., gallbladder corpus.

* , *p* < 0.05;

** , *p* < 0.01;

*** , *p* < 0.001 (Statistical significances versus relevant ‘non-fed’ value).

† , *p* < 0.05 (Statistical significances versus relevant ‘fast’ value).

§ , *p* < 0.001.

The duration of the second (longer) fragment of LSBP was only slightly shorter than the total duration of the pattern, and the changes related to the various feeding conditions were similar (Table 1 and Table 4). The frequency of the spikes forming this fragment was much lower than that of its first part, and it was higher in non-fasted animals than in fasted animals (Table 3 and Table 4). The number of spikes forming this part of the LSBP was smaller in fasted rams than in the remaining groups of the experiments (Table 4).

**TABLE 4:** Characteristics of the longer fragment of the long spike burst pattern recorded in the gallbladder infundibulum and corpus in various feeding conditions.

Fasting	Feeding	Mean ± S.D.	Duration (s)		Spike frequency (No/s)		Spike (*N*)	
			Infund.	Corp.	Infund.	Corp.	Infund.	Corp.
Fasted	Not fed	Mean	54.9	16.1	1.3	1.4	24.3	18.2
		± SD	13.3	6.9	0.2	0.3	5.9	3.4
	Fed	Mean	23.2*	10.1	1.5	1.3	35.8*	25.4*
		± SD	7.8	4.2	0.3	0.4	6.4	4.2
Not fasted	Not fed	Mean	30.2‡	12.8	2.0†	1.6	32.6	27.3
		± SD	7.5	7.1	0.6	0.5	5.2	3.9
	Fed	Mean	15.4**	7.8	2.2†	1.8	31.4	26.0
		± SD	4.4	6.2	0.5	0.4	9.8	4.4

Note: Other explanations are the same as in Tables 1 and 2, and also presented in the ‘Materials and methods’ section.

LSBP, long spike burst pattern; Spikes (N), number of spikes in the whole LSBP; infund., gallbladder infundibulum; corp., gallbladder corpus.

* , *p* < 0.05;

** , *p* < 0.01 (Statistical significances versus relevant ‘not fed’ value).

† , *p* < 0.05,

‡ , *p* < 0.01 (Statistical significances versus relevant ‘fast’ value).

The incidence of longer or shorter phasic contraction of the gallbladder fundus, recorded with the strain gauge method, followed in almost all cases the spike bursts. In fasted rams, the duration and frequency of longer contractions of the gallbladder infundibulum were significantly greater in fed animals than in non-fed animals (Table 5). The amplitude was not significantly higher in fed rams as compared with non-fed rams. The duration of the shorter contractions was significantly reduced in fed rams, whereas both the amplitude and frequency were not significantly lower in fed animals than in non-fed animals. In non-fasted rams, the duration of longer contractions of the gallbladder fundus was significantly longer in fed animals than in non-fed animals and was also significantly prolonged as compared with the corresponding value obtained in fasted rams. The amplitude of longer contractions was slightly higher in fasted rams after feeding compared to non-fed rams. The frequency of longer contractions was slightly but significantly higher in non-fasted non-fed than in fasted non-fed rams. The duration of shorter contractions in non-fasted fed rams was significantly shorter than in non-fasted non-fed animals. The amplitude of shorter contractions in fasted rams was slightly lower after feeding compared to non-fasted animals. The frequency of shorter contractions in non-fasted non-fed rams was not significantly higher than in non-fasted fed animals (Table 5).

**TABLE 5:** Characteristics of the longer and shorter gallbladder fundic contractions recorded during phase 2-like activity of the putative gallbladder migrating myoelectric complex in various feeding conditions.

Fasting	Feeding	Mean ± S.D.	Longer contractions			Shorter contractions		
			Duration (s)	Amplitude (g)	Frequency (cpm)	Duration (s)	Amplitude (g)	Frequency (cpm)
Fasted	Non-fed	Mean	15.4	8.8	0.3	4.8	1.8	17.3
		± SD	4.6	5.4	0.1	0.4	0.5	6.1
	Fed	Mean	26.3*	14.8	0.8**	3.4***	1.2	12.9
		± SD	3.7	7.9	0.3	0.3	0.4	6.1
Non-fasted	Non-fed	Mean	16.5	11.2	0.5†	4.5	1.6	15.2
		± SD	5.2	6.5	0.3	0.6	0.7	4.1
	Fed	Mean	38.7**†	14.4	0.8	3.1***	1.8	12.2
		± SD	10.3	8.8	0.5	0.2	0.6	5.8

Note: Other explanations are the same as in Table 1 and also presented in the ‘Materials and methods’ section.

*n* = 4.

s, second; g, gram; cpm, cycles per minute.

* , *p* < 0.05;

** , *p* < 0.01;

*** , *p* < 0.001 (Statistical significances versus relevant ‘non-fed’ value).

† , *p* < 0.05 (Statistical significances versus relevant ‘fast’ value).

## Discussion

The results obtained in this study characterise in part the gallbladder myoelectrical activity with some regional differences. Compared with the small-intestinal motility, it exhibits similarities and differences as it was initially reported earlier (Roman´ski 1996, 2002b, 2004a). The MMC presented the principal pattern occurring in the ovine small bowel regardless of feeding conditions and a similar pattern appeared to occur also in the gallbladder. The duration and regularity of the spike bursts are similar in the small bowel and gallbladder, whereas the occurrence of the unique pattern in the upper gallbladder (LSBP) was specific for this organ.

The shape, duration and spike frequency of the short-lasting spike bursts in the ovine gallbladder (representing the myoelectrical correlates of shorter phasic contractions) appeared not to be markedly different from those in the small bowel, but their amplitude was relatively low. This results from the thinner smooth muscle layer of the gallbladder wall as compared with that in the small bowel. Not only the amplitude of the spike bursts but also the force of phasic contractions seemed to be smaller in the gallbladder than in the small bowel as was observed in the present and previous studies (Grivel & Ruckebusch 1972; Roman´ski 2003). In the gastrointestinal tract, feeding evokes the overall increase of the myoelectric and contractile activity while suppressing the spike bursts and contraction amplitudes (Heddle, Miedema & Kelly 1993; Lester & Bolton 1994; Roman´ski 2003; Wingate et al. 1979). A similar response to feeding was also observed in this study in the ovine gallbladder.

Long spike burst pattern is the novel event occurring in the upper ovine gallbladder. The first part was very similar to the typical but slightly longer spike burst, representing the myoelectrical correlate of phasic contraction. This part always occurred at the beginning of LSBP both in the gallbladder infundibulum and gallbladder corpus. As this part could appear twice within one LSBP and occurred sometimes in the various sites of the longer part of the pattern, it seems likely that the same typical spike bursts are superimposed on the LSBP arriving during this pattern. Its second part was different because the spikes were less frequent than in the first part and their shape slightly differed from the typical spikes. During the second part of the LSBP, spikes occurred at a much lower frequency than during the first part, implying a different characteristic. Similar events were also shown earlier (Buéno & Praddaude 1979; see also Roman´ski 2004a). The slow waves were observed in the gallbladder infrequently. According to the suggestion of Matsumoto, Sarna and Condon (1985) and of Becker, Duff and Moody (1981), the slow waves can occur in canine and opossum gallbladder and are omnipresent there. In ovine gallbladder, they occurred at much lower frequency than the spikes observed within the second part of the LSBP (see also Roman´ski 2004a). Therefore, it is likely that LSBPs contained only the spikes, not the slow waves.

The question regarding the physiological role of LSBP is open. It is possible that it controls the episodes of gallbladder filling and emptying. Relaxation and lowering of the pressure in the upper gallbladder region (pressure changes in ovine gallbladder are quite frequent, Nejmark 1977) is necessary for gallbladder filling. Soon after the pressure may rise because of the occurrence of a contraction induced at least by the first part of LSBP to evacuate a small portion of bile. The subsequent arrival of the second, longer part of LSBP increases tension in this region, which prevents bile inflow to the gallbladder from the common bile duct. It can occur especially when the amplitude of spikes forming the LSBP is high. This repeatable mechanism might be responsible for undisturbed bile inflow and outflow in cooperation with the sphincters located in the biliary tract, that is, Mirizzi’s sphincter, Lütkens’ sphincter and mainly the Oddi’s sphincter (Bagcivan et al. 2006; Grivell et al. 2004; Levina 1971; Mirizzi 1940; see also Nejmark 1977). The role of Mirizzi’s and Lütkens’ sphincters is difficult to assess because they represent the functional sphincters only. Bile circulation between the liver, gallbladder and duodenum occurs during the interdigestive state and is more intense after a meal (Roman´ski 2004b; Ruckebusch 1989; Scott & Diamant 1988). It might be slightly increased because of the presence of the duct of Luschka (Spanos & Syrakos 2006), but it is not known whether or not this duct occurs in sheep. The arrival of the longer LSBPs, especially in fasted sheep, might help in reducing bile outflow during this period, facilitating bile storage and concentration. Intensified LSBPs after feeding might be responsible for bile evacuation, at least in part.

Different types of spike bursts, concerning their duration and amplitude, were found in the ovine gallbladder, and the question arises as to which types of contractions can be evoked by these spike bursts. This is also a question of terminology. According to the duration criterion, two types of contractions – phasic and tonic – can be distinguished. No myoelectric correlates of tonic contractions have been described. Assuming that tonic contractions last several minutes or more (Sarna 2002), it can be stated that the duration of LSBPs and of other longer spike bursts arriving both in the gallbladder and small intestine, for example, the giant spike bursts, is too short to evoke tonic contractions. Therefore, the contractions observed in the ovine gallbladder, lasting 5–10 sec, or even longer, should not be called tonic contractions. These contractions and their myoelectrical correlates, that is, giant-like contractions or giant-like spike bursts, respectively, can form specific patterns as LSBP in the gallbladder, but also similar events can be present in the small or large bowel (Fioramonti & Hubert 1980; Sarna 2002). These longer spike bursts cannot probably be evoked by the slow waves because they last longer than their plateau phase. According to the definition of tonic contractions proposed by Sarna (2002), any contractions that are shorter than several minutes could be classified as phasic contractions. However, this term has not yet been precisely defined.

The applied electromyographical technique with inert teflon-platinum electrodes is apparently not harmful and yet sensitive enough to detect all the myoelectrical events in the normal gallbladder. However, not all the events, especially the slow waves, were regularly recorded here. Therefore, it can be assumed that in the ovine gallbladder, the slow waves occur infrequently or their amplitude can be occasionally reduced to an undetectable level. Some authors found neither slow waves nor spiking activity in the gallbladder of the dog or monkey. It may have been that techniques applied could explain this failure (Becker et al. 1981; Ludwick & Bass 1967). Thus, sheep may represent the suitable model for recording of the gallbladder myoelectrical activity. The spiking activity in the gallbladder was found also in the dog (Itoh et al. 1982; Traynor, Dozois & Dimagno 1984; Ura, Sarna & Condon 1992). Satisfactory recordings of the gallbladder electrical activity were obtained also in pigs (Laplace 1976a, 1976b) and later in sheep (Buéno & Praddaude 1979). These scanty data suggest that application of the technique of recording of the gallbladder myoelectrical activity in sheep and in other species is possible, but also interpretation of the results can be problematic.

Taken together, it is clear that the intense and composed myoelectrical and motor activity occurs permanently in the ovine gallbladder. The gallbladder exhibits also specific LSBP. The character of an LSBP is dependent on the gallbladder region and feeding conditions. Feeding exerts marked alterations in the gallbladder spike bursts, mostly lowering their amplitude and prolonging the duration.

## References

[CIT0001] Aziz S.H. & Khatra G.S., 1985, ‘Quantitative morphological study on liver and gall bladder of sheep’, *Journal of Research in Punjab Agricultural University* 22(6), 753–756.

[CIT0002] Bagcivan I., Kaya T., Yidirim M.K. & Turan M., 2006, ‘Investigation of the relaxant effects of pinacidil and cromakalim on the sheep sphincter of Oddi’, *Pancreatology* 6(4), 286–290. https://doi.org/10.1159/000092690 16636601 10.1159/000092690

[CIT0003] Becker J.M., Duff W.M. & Moody F.G., 1981, ‘Myoelectric control of gastrointestinal and biliary motility: A review’, *Surgery* 89(4), 466–477. 7010653

[CIT0004] Buéno L. & Praddaude F., 1979, ‘Electrical activity of the gallbladder and biliary tract in sheep and its relationships with antral and duodenal motility’, *Annals of Biology of Animals and Biochemistry and Biophysics* 19(10), 1109–1121. https://doi.org/10.1051/rnd:19790711

[CIT0005] Dodds W.Y., Hogan W.J. & Geenen J.E., 1989, ‘Motility of the biliary system’, in Schultz S.G. (ed.), *Handbook of physiology. The gastrointestinal system*, pp. 1055–1101, American Physiological Society, Bethesda, MD.

[CIT0006] Fioramonti J. & Hubert M.F., 1980, ‘Motor function of the large intestine in sheep *versus* cattle’, *Annales de la Recherche Vétérinaire* 11(1), 109–115. 7436326

[CIT0007] Grivel M.L. & Ruckebusch Y., 1972, ‘The propagation of segmental contractions along the small intestine’, *Journal of Physiology (London)* 227, 611–625. https://doi.org/10.1113/jphysiol.1972.sp010050 4647272 10.1113/jphysiol.1972.sp010050 PMC1331213

[CIT0008] Grivell M.B., Woods C.M., Grivell A.R., Neild T.O., Craig A.G., Toouli J. et al., 2004, ‘The possum sphincter of Oddi pumps or resists flow depending on common bile duct pressure: A multilumen manometry study’, *Journal of Physiology (London)* 558(2), 611–622. https://doi.org/10.1113/jphysiol.2004.061663 15169843 10.1113/jphysiol.2004.061663 PMC1664969

[CIT0009] Heddle R., Miedema B.W. & Kelly K.A., 1993, ‘Integration of canine proximal gastric, antral, pyloric, and proximal duodenal motility during fasting and after a liquid meal’, *Digestive Diseases and Sciences* 38(5), 856–869. https://doi.org/10.1007/BF01295912 8482185 10.1007/BF01295912

[CIT0010] Itoh Z., Takahashi I., Nakaya M., Suzuki T., Arai H. & Wakabayashi K., 1982, ‘Interdigestive gallbladder bile concentration in relation to periodic contraction of gallbladder in the dog’, *Gastroenterology* 83(3), 645–651. 7095368

[CIT0011] Kaji T., Takamatsu H. & Kojiya H., 2002, ‘Motility of the gastrointestinal tract and gallbladder during long-term total parenteral nutrition in dogs’, *Journal of Parenteral and Enteral Nutrition* 26(3), 198–204. https://doi.org/10.1177/0148607102026003198 12005462 10.1177/0148607102026003198

[CIT0012] Laplace J.P., 1976a, ‘L’excrétion biliaire chez le Porc. 1) Électromyographie des voies biliaires extrahépatiques’, *Recoil de Médecine Vétérinaire* 152(1), 33–43.

[CIT0013] Laplace J.P., 1976b, ‘L’excrétion biliaire chez le Porc. 2) Électromyographie et dynamique de l’excrétion de bile’, *Recoil de Médecine Vétérinaire* 152(4), 401–411.

[CIT0014] Lee S.P. & Kuwer R., 2006, ‘Gallbladder function’, in Johnson L.R. (ed.), *Physiology of the gastrointestinal tract*, pp. 1535–1557, Elsevier, Inc., Amsterdam.

[CIT0015] Lester G.D. & Bolton J.R., 1994, ‘Effect of dietary composition on abomasal and duodenal myoelectrical activity’, *Research in Veterinary Sciences* 57(3), 270–276. https://doi.org/10.1016/0034-5288(94)90117-1 10.1016/0034-5288(94)90117-1 7871244

[CIT0016] Levina S.I., 1971, ‘The genesis of Lutkens’ sphincter in the light of new findings concerning the structure of the gall bladder wall in relation to age’, *Arkhivna Patologia* 33(10), 58–64. 5143335

[CIT0017] Ludwick J.R. & Bass P., 1967, ‘Contractile and electric activity of the extrahepatic biliary tract and duodenum’, *Surgery, Gynecology and Obstetrics* 124(3), 536–546. 4959863

[CIT0018] Matsumoto T., Sarna S.K. & Condon R.E., 1985, ‘Gallbladder electrical activity in vivo’, *Gastroenterology* 88, 1493 (abstr.).

[CIT0019] Mirizzi P.L., 1940, ‘Physiologic sphincter of hepatic bile duct’, *Archives of Surgery* 41, 1325–1333. https://doi.org/10.1001/archsurg.1940.01210060022003

[CIT0020] Nejmark L., 1977, ‘The functions of bile ducts in sheep’, *Acta Physiologica Polonica* 28(5), 463–474. 596195

[CIT0021] Pass M.A. & Heath T.J., 1977, ‘Factors affecting gallbladder motility in sheep’, *Comparative Biochemistry and Physiology C* 56(2), 127–131. https://doi.org/10.1016/0306-4492(77)90027-2 10.1016/0306-4492(77)90027-2 15772

[CIT0022] Romański K.W., 1996, ‘The myoelectric (M) patterns in ovine gallbladder (GB)’, *Journal of Physiology and Pharmacology* 47(Suppl. 2), 102 (abstr.).

[CIT0023] Romański K.W., 2002a, ‘Influence of various feeding conditiona, the migrating myoelectric complex and cholinergic drugs on antral slow waves in sheep’, *Archives of Animal Nutrition* 56(3), 393–408. 12553690 10.1080/00039420215638

[CIT0024] Romański K.W., 2002b, ‘Characteristics and cholinergic control of the “minute rhythm” in ovine antrum, small bowel and gallbladder’, *Journal of Veterinary Medicine A* 49(6), 313–320. https://doi.org/10.1046/j.1439-0442.2002.00399.x 10.1046/j.1439-0442.2002.00399.x 12227475

[CIT0025] Romański K.W., 2003, ‘Character and cholinergic control of myoelectric activity in duodenal bulb: Relationships to adjacent regions’, *Veterinarski Arhiv* 73(1), 1–16.

[CIT0026] Romański K.W., 2004a, ‘Ovine model for clear-cut study on the role of cholecystokinin in antral, small intestinal and gallbladder motility’, *Polish Journal of Pharmacology* 56(2), 247–256. 15156076

[CIT0027] Romański K.W., 2004b, ‘Feeding versus cholecystokinin – Spectrum of actions on ovine gallbladder contractility assessed with real-time ultrasonography’, *Wiener Tierärztliche Monatsschrift* 91(9), 226–235.

[CIT0028] Romański K.W., 2016, ‘The diversity of “minute rhythm” forms in the ovine small bowel: Relationship to feeding and to the phase of the migrating myoelectric complex’, *Veterinarski Arhiv* 86(3), 351–362.

[CIT0029] Romański K.W., 2017, ‘Occurrence and characteristics of the migrating myoelectric complex in ovine gallbladder and its relationships to the small intestinal motility’, *European Journal of Biological Research* 7(2), 139–147.

[CIT0030] Ruckebusch Y., 1989, ‘Gastrointestinal motor function in ruminants’, in Schultz S.G. (ed.), *Handbook of physiology. The gastrointestinal system*, pp. 1225–1282, American Physiological Society, Bethesda, MD.

[CIT0031] Ryan J.P., 1981, ‘Motility of the gallbladder and biliary tree’, in Johnson L.R. (ed.), *Physiology of the gastrointestinal tract*, pp. 473–494, Raven Press, New York.

[CIT0032] Sanders K.M., Ward S.M. & Koh S.D., 2014, ‘Interstitial cells: Regulators of smooth muscle function’, *Physiological Review* 94(3), 859–907. https://doi.org/10.1152/physrev.00037.2013 10.1152/physrev.00037.2013 PMC4152167 24987007

[CIT0033] Sarna S.K., 2002, ‘Myoelectrical and contractile activities of the gastrointestinal tract’, in Schuster M.M., Crowell M.D. & Koch K.L. (eds.), *Schuster atlas of gastrointestinal motility in health and disease*, pp. 1–18, B.C. Decker, Inc., Hamilton.

[CIT0034] Scott R.B. & Diamant N.E., 1988, ‘Biliary motility associated with gallbladder storage and duodenal delivery of canine hepatic biliary output’, *Gastroenterology* 95(4), 1069–1080. https://doi.org/10.1016/0016-5085(88)90185-0 3410222 10.1016/0016-5085(88)90185-0

[CIT0035] Shaffer E.A., 2000, ‘Review article: Control of gall-bladder motor function’, *Alimentary Pharmacology and Therapy* 14(Suppl. 2), 2–8. https://doi.org/10.1046/j.1365-2036.2000.014s2002.x 10.1046/j.1365-2036.2000.014s2002.x 10902995

[CIT0036] Snedecor G.W. & Cochran W.G., 1971, *Statistical methods*, The Iowa State University Press, Ames, IO.

[CIT0037] Spanos C.P. & Syrakos T., 2006, ‘Bile leaks from the duct of Luschka (subvesical duct): A review’, *Langenbecks Archives of Surgery* 391(5), 441–447. https://doi.org/10.1007/s00423-006-0078-9 10.1007/s00423-006-0078-9 16927110

[CIT0038] Szurszewski J.H., 1987, ‘Electrical basis for gastrointestinal motility’, in Johnson L.R. (ed.), *Physiology of the gastrointestinal tract*, pp. 383–422, Raven Press, New York.

[CIT0039] Thuneberg L., 1999, ‘One hundred years of interstitial cells of Cajal’, *Microscopic Research Techniques* 47(2), 223–238. https://doi.org/10.1002/(SICI)1097-0029(19991115)47:4%3C223::AID-JEMT2%3E3.0.CO;2-C 10.1002/(SICI)1097-0029(19991115)47:4<223::AID-JEMT2>3.0.CO;2-C 10602284

[CIT0040] Traynor O.J., Dozois R.R. & Dimagno E.P., 1984, ‘Canine interdigestive and postprandial gallbladder motility and emptying’, *American Journal of Physiology* 246(4 Pt 1), G426–G432. https://doi.org/10.1152/ajpgi.1984.246.4.G426 6720894 10.1152/ajpgi.1984.246.4.G426

[CIT0041] Ura K., Sarna S.K. & Condon R.E., 1992, ‘Antral control of gallbladder cyclic motor activity in the fasting state’, *Gastroenterology* 102(1), 295–302. https://doi.org/10.1016/0016-5085(92)91813-J 1727761 10.1016/0016-5085(92)91813-j

[CIT0042] Wingate D., Pierce E., Ling A., Boucher B., Thompson H. & Hutton M., 1979, ‘Quantitative effect of oral feeding on gastrointestinal myoelectric activity in the conscious dogs’, *Digestive Diseases and Sciences* 24(6), 417–423. https://doi.org/10.1007/BF01299823 456227 10.1007/BF01299823

